# Factors Associated With Pediatric Emergency Airway Management by the Difficult Airway Response Team

**DOI:** 10.7759/cureus.16118

**Published:** 2021-07-02

**Authors:** Nicholas M Dalesio, Lauren Burgunder, Natalia M Diaz-Rodriguez, Sara I Jones, Jordan Duval-Arnould, Laeben C Lester, David E Tunkel, Sapna R Kudchadkar

**Affiliations:** 1 Otolaryngology/Head & Neck Surgery, Johns Hopkins University School of Medicine, Baltimore, USA; 2 Anesthesiology and Critical Care, Johns Hopkins University School of Medicine, Baltimore, USA; 3 Pediatrics, Johns Hopkins University School of Medicine, Baltimore, USA; 4 Johns Hopkins Medicine Simulation Center, Johns Hopkins University School of Medicine, Baltimore, USA

**Keywords:** airway disorders, difficult airway management, pediatric anesthesiology, interests in difficult airway and regional anaesthesia, multi-disciplinary teams, rapid response teams

## Abstract

Background

The goal of this study was to determine if difficult airway risk factors were similar in children cared for by the difficult airway response team (DART) and those cared for by the rapid response team (RRT).

Methods

In this retrospective database analysis of prospectively collected data, we analyzed patient demographics, comorbidities, history of difficult intubation, and intubation event details, including time and place of the emergency and devices used to successfully secure the airway.

Results

Within the 110-patient cohort, median age (IQR) was higher among DART patients than among RRT patients [8.5 years (0.9-14.6) versus 0.3 years (0.04-3.6); P* *< 0.001]. The odds of DART management were higher for children ages 1-2 years (aOR, 43.3; 95% CI: 2.73-684.3) and >5 years (aOR, 13.1; 95% CI: 1.85-93.4) than for those less than one-year-old. DART patients were more likely to have craniofacial abnormalities (aOR, 51.6; 95% CI: 2.50-1065.1), airway swelling (aOR, 240.1; 95% CI: 13.6-4237.2), or trauma (all DART managed). Among patients intubated by the DART, children with a history of difficult airway were more likely to have musculoskeletal (P* *= 0.04) and craniofacial abnormalities (P < 0.001), whereas children without a known history of difficult airway were more likely to have airway swelling (P = 0.04).

Conclusion

Specific clinical risk factors predict the need for emergency airway management by the DART in the pediatric hospital setting. The coordinated use of a DART to respond to difficult airway emergencies may limit attempts at endotracheal tube placement and mitigate morbidity.

## Introduction

Difficult airways in children are rare; however, airway-related complications are a significant cause of morbidity and mortality. In the pediatric operating suite (OR), approximately 0.02% of cases involve difficult mask ventilation and 0.25% to 0.32% of cases have difficult intubation [1,2]. A 2014 publication reported that outside the OR, in the pediatric intensive care unit (PICU), approximately 9% of all tracheal intubations could be classified as difficult, requiring three or more attempts [3]. Senior-level practitioners performed most of the initial intubation attempts in such cases (81%), yet severe adverse events, including cardiac arrest, esophageal intubation with delayed recognition, and emesis with witnessed aspiration, remained high, at 13%.

Both the Joint Commission on Accreditation of Healthcare Organizations and the Institute for Healthcare Improvement via the 100,000 Lives Campaign recommend that hospitals have “a system of rapid response teams (RRTs) to bring skilled resources” [4]. Most hospitals have RRTs for first-line assessment and management of patients with acute clinical deterioration. At our institution, the pediatric RRT consists of an intensivist, a PICU fellow, a respiratory therapist, and a pediatric nurse, and intubation is performed by the in-house PICU fellow or attending. However, a team of airway experts can provide critical support and personnel proficient in advanced airway management devices and techniques not routinely used outside the OR. Therefore, in 2008, our institution created the difficult airway response team (DART) composed of anesthesiologists, intensivists, nurses, respiratory therapists, otolaryngology-head and neck surgeons, and trauma surgeons, to manage adults and children with a difficult airway in inpatient settings [5]. The DART team is routinely called if (1) a patient with history of difficult airway is in respiratory distress, (2) multiple attempts at securing the airway have failed, or (3) difficulty securing the airway is likely, based on the mechanisms for respiratory compromise. 

Difficulties in pediatric airway management can often be predicted by the presence of specific risk factors identified via medical history and physical examination [6,7]. However, specific patient and contextual factors (e.g., time of day, location) associated with airway management by the DART have not been described in the pediatric inpatient setting. Thus, we sought to compare demographic, clinical, and situational risk factors of pediatric inpatients who were successfully intubated by the RRT to those who required the DART at our tertiary-care hospital. 

## Materials and methods

The institutional review board (IRB) at our university approved the study protocol (IRB# NA_00089582) and waived the requirement for informed consent. 

Study design

**Figure 1:**
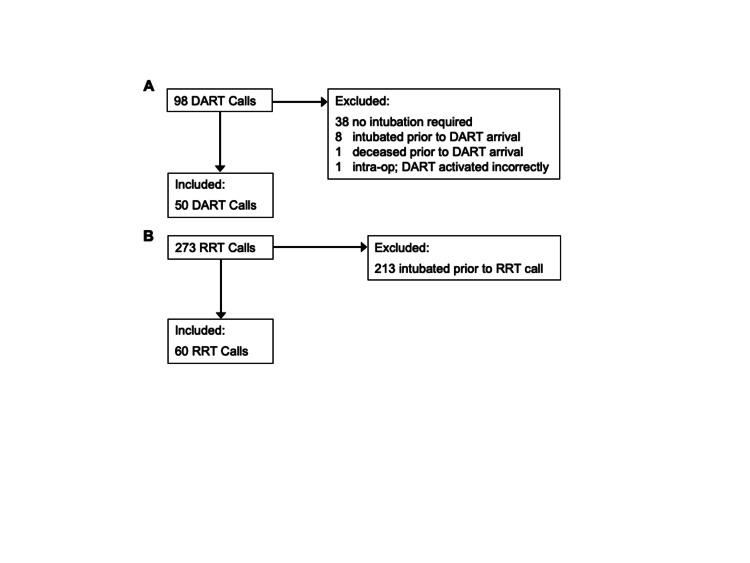
Flow chart for inclusion and exclusion of (A) difficult airway response team (DART) and (B) rapid response team (RRT) cases.

We conducted a retrospective database analysis of prospectively collected data from the DART registry between February 1, 2009, and December 31, 2015. The DART registry is a prospectively collected database of all DART calls in which the responding provider enters demographic data and details regarding airway management during the DART response. Patients were included if they were less than 15 years of age or were cared for in the children’s center. We compared characteristics of children intubated by the DART to those of children in the Resuscitation Event Analysis Clearinghouse (REACH) Surveillance System [8] who were intubated by the RRT between January 24, 2013, and March 14, 2016. If the RRT had difficulty securing the airway of a patient, and consequently activated the DART for intervention, we included that patient in the DART dataset (Figure 1). Patients with a known history of difficult airway had initial airway management performed by the DART. 

We collected demographic data including patient age, weight, history of difficult airway, and comorbid conditions. Information about airway pathology, including airway bleeding and trauma, was also collected. Intubation devices and attempts, patient location (e.g., inpatient floor, emergency department [ED], PICU, and neonatal intensive care unit [NICU]), whether cardiopulmonary resuscitation (CPR) was underway during intubation, and provider training level were also noted. In several situations, efforts were made to mobilize pediatric DART patients to the OR for use of advanced airway management techniques and devices.

Statistical analysis

Categorical data were analyzed by chi-squared test, and continuous data (expressed as medians with interquartile range [IQR]) were analyzed by the Mann-Whitney U-test. We applied univariate analysis as well as multivariable logistic regression modeling to determine the differences in patient comorbidities and intubation scenarios between the groups. A P-value < 0.05 was considered statistically significant. Stata SE 15.1 (StataCorp, College Station, TX) was used to conduct the statistical analysis.

## Results

Demographics

A total of 110 patients (45% female) were eligible for inclusion in the analysis. Sixty patients were intubated by the RRT (54.5%), and the remaining 50 patients had intervention by the DART. In the latter group, 29 (58%) had a history of difficult airway, and 21 (42%) had unanticipated difficult airway. Three patients had more than two DART calls, either during one or multiple hospitalizations. As shown in Table 1, children in the RRT group were significantly younger (median age 0.3 years [IQR, 0.04-3.6]) than children intubated by the DART (median age 8.5 years [IQR, 0.9-14.6]; P < 0.001). 

**Table 1:** Demographic data of patients intubated by the rapid response team and those intubated by the difficult airway response team. DART: difficult airway response team; IQR: interquartile range; RRT: rapid response team. ^a^Statistical differences were assessed with the Mann-Whitney test for nonparametric continuous data and with the Pearson’s chi-squared test for categorical variables. ^b^Weight was missing for one patient in the RRT group.

Characteristic	Total (n = 110)	RRT intubation (n = 60)	DART intubation (n = 50)	P-value^a^
Age, years, median (IQR)	1.1 (0.1–10.6)	0.3 (0.04–3.6)	8.5 (0.9–14.6)	< 0.001
Age category, n (%)				< 0.001
<1 year	51 (46)	38 (63)	13 (26)	
1–2 years	11 (10)	3 (5)	8 (16)	
2–5 years	12 (11)	9 (15)	3 (6)	
>5 years	36 (33)	10 (17)	26 (52)	
Sex, n (%)				0.15
Male	60 (55)	29 (48)	31 (62)	
Female	50 (45)	31 (52)	19 (38)	
Weight, kg, median (IQR)^b^	9.4 (3.2–29.7)	4.2 (1.7–12.9)	18.4 (7.5–57)	< 0.001

Location of airway management

The most common location for intubations by both RRT (65%) and DART (70%) were the intensive care units (ICUs). RRTs occurred more commonly in the NICU, whereas DARTs occurred more commonly in the PICU (RRT: 42% NICU, 23% PICU; DART: 8% NICU, 62% PICU). RRT and DART had similar percentages of response calls to the ED (18% and 22%, respectively, P = 0.54). Both RRT- and DART-led intubations took place in the OR only when patients were transferred there emergently from another location for use of advanced airway management techniques and equipment stored there. Patients located on the inpatient floor were more likely to be successfully cared for by the RRT and not require the DART (n = 5 RRT; n = 1 DART; Table 2).

**Table 2:** Comparison of patient groups that underwent invasive airway management by the rapid response team (RRT) and by the difficult airway response team (DART). DART: difficult airway response team; ICU: intensive care unit; NA: not applicable; PACU: post-anesthesia care unit; CPR: cardiopulmonary resuscitation; RRT: rapid response team; RSI: rapid sequence intubation; SGA: supraglottic airway. ^a^Statistical differences were assessed by the Mann-Whitney test for nonparametric data for continuous variables and Pearson’s chi-squared test for categorical variables. ^b^Efforts are made to move patients to the operating room suite if advanced airway techniques are needed (e.g., fiberoptic bronchoscopes, inhalational anesthesia). DART calls are not made on patients already in the operating suite. ^c^Eight DART patients did not have a time of day registered. ^d^One patient had care withdrawn after intubation attempts. ^e^Attempts before DART activation were not recorded for two patients. All patients with history of difficult airway had initial intubation attempts performed by the DART. ^f^Rigid laryngoscopy is defined as intubation by the otolaryngologist using operative laryngoscopes or a rigid bronchoscope.

Characteristic	Total (n = 110)	RRT intubation (n = 60)	DART intubation (n = 50)	P-value^a^
Intubation location, n (%)				0.54
ICU	73 (66)	39 (65)	35 (62)	
Medical floor	6 (6)	5 (8)	1 (2)	
Operating suite^b^	6 (5)	4 (7)	3 (6)	
PACU	1 (1)	1 (2)	0	
Emergency department	24 (22)	11 (18)	11 (22)	
Time of day, n (%)^c^	102	60 (59)	42 (41)	0.04
6 am–12 pm	38 (37)	14 (23)	24 (57)	
12 pm–6 pm	20 (20)	15 (25)	4 (10)	
6 pm–12 am	16 (16)	16 (27)	1 (2)	
12 am–6 am	28 (27)	15 (25)	13 (31)	
Intubation attempts^d^				
Median (IQR)	1 (1-3)	1 (1-5)	2.5 (1-4)	<0.001
Before DART call^e^		NA	0 (0-1.5)	
After DART call		NA	1 (1-2.5)	
Rank of successful clinician, n (%)				0.25
Attending	29	13 (22)	16 (32)	
Fellow	19	13 (22)	6 (12)	
Resident or nurse practitioner	3	2 (3)	1 (2)	
Not recorded	59	32 (53)	27 (54)	
Intubation techniques attempted				<0.001
Direct laryngoscopy with RSI	64	59	5	
Direct laryngoscopy without RSI	9	0	9	
Videolaryngoscopy (GlideScope® or CMAC®)	7	1	6	
Fiberoptic intubation	6	0	6	
Fiberoptic intubation via SGA	5	0	5	
Rigid laryngoscopy^f^	12	0	12	
Surgical airway^g^	7	0	7	

Time of day for RRT and DART activation

Over half of DART calls with a known time of day (24 of 42) occurred between 6 am and 12 pm, compared with only 23% (14 of 60) of RRT calls (P = 0.04). The second most common time for DART activation was during late-night hours between 12 am and 6 am (31%; Table 2).

Intubation attempts

Of the 60 RRT patients, 44 (73%) were intubated after one attempt and 52 (87%) had a secure airway within three attempts. Eight RRT patients (13%) required more than three attempts to secure the airway, of which, five were neonates under 1 month of age and one had a craniofacial abnormality. One patient required up to six attempts (Table 2).

The median number of attempts to secure the airway in DART patients was 2.5 (IQR, 1-4), and 74% had their airway secured within three attempts. In DART activations where the difficult airway was not anticipated, the ED or ICU staff performed between three and six intubation attempts; however, intubation attempts prior to DART activation were not documented in two of the 50 patients. Three patients required six attempts and one patient required 10 attempts (six attempts prior to DART activation and four attempts by the DART) to secure the airway. After the DART responded, 27 of 48 (56.3%) were intubated on the first attempt, and 44 of 48 (91.7%) were intubated in three or fewer attempts (Table 2). One DART patient with an unanticipated difficult airway died during intubation in a “cannot ventilate, cannot intubate” scenario. No patients died during intubation by the RRT. 

The physician’s experience and seniority of the clinician who secured the airway were not consistently recorded and were missing in 32 of the 60 (53.3%) RRT records and 27 of the 50 (54.0%) DART records. Among complete records, the attending or fellow successfully intubated 26 of the 28 (92.9%) patients in the RRT group and 22 of the 23 (95.7%) patients in the DART group (Table 2). 

Intubation techniques

Almost all of the RRT intubations were performed by direct laryngoscopy with a rapid sequence induction (59 of 60). One patient in the ICU who required intubation because of blood in the airway was intubated after one attempt by the RRT using a videolaryngoscope. Thirty-six of the 50 DART patients (72.0%) required an advanced technique for intubation. Twelve (33.3%) of those who required advanced techniques were successfully intubated by an otolaryngologist using operative laryngoscopes with or without a rigid bronchoscope, and 11 (30.6%) required fiberoptic intubation either transorally or via a supraglottic airway. Six surgical airways were performed. One was a new tracheostomy in an 18-year-old who had asthma and subglottic stenosis likely caused by prolonged intubation. A 14-year-old patient received a needle cricothyrotomy, and four patients received revision tracheostomies (Table 2).

Risk factors

Most DART patients (84%) had at least one difficult airway risk factor (Table 3) compared to only 27% (16 of 60) of RRT patients. In unadjusted analysis, the presence of one or more clinical difficult airway risk factors, such as bleeding, trauma, edema, difficult airway history, or musculoskeletal and craniofacial abnormality was highly significantly associated with intubation by the DART (odds ratio, 14.4; 95% CI: 5.6-37.3). It was less common for patients undergoing CPR to require the DART for intubation (OR, 0.09; 95% CI: 0.04-0.22; Table 3) 

**Table 3:** Univariate and multivariable adjusted odds ratios for comorbidities comparing patients with difficult intubation performed by the Difficult Airway Response Team and those undergoing intubation by the rapid response team aOR: adjusted odds ratio; CI: confidence interval; CPR: cardiopulmonary resuscitation; DART: difficult airway response team; NA: not applicable; OR: odds ratio. ^a^Difficult airway history indicates patients who had been diagnosed by an anesthesiologist in the past. Airway bleeding includes patients with post-tonsillectomy bleeding and airway trauma.  Airway swelling includes patients with anaphylaxis, airway burns or inhalational injury, subglottic stenosis, and airway trauma.  Trauma includes patients who had been in a motor vehicle crash, had attempted suicide, or had a gunshot wound. Genetic syndromes include Algille’s, Epidermolysis bullosa, and Moya Moya disease. Craniofacial diseases include Treacher-Collins, Pierre-Robin, Noonan’s, and Hunter’s syndrome. Musculoskeletal diseases include muscular dystrophy. ^b^Crude estimates without adjustment for any covariate. ^c^Statistical differences were assessed with multivariable logistic regression modeling. ^d^Adjusted estimate includes all variables in the table except for the subgroups “Multiple risk factors” and “History of difficult airway” because all of these patients automatically have the DART called for any respiratory emergency. ^e^All patients were cared for by the DART. ^f^Craniofacial conditions include conditions that are caused by embryologic etiologies as well as those caused by genetic mutations. Patients with genetic etiologies for their craniofacial condition were included in both genetic and craniofacial analyses.

Comorbidity^a^	Total	Crude OR (95% CI)^b^	P-value^c^	aOR (95% CI)^d^	P-value^c^
Age					
<1 year	51	1 (reference)	-	1 (reference)	-
1–2 years	11	7.8 (1.79-33.9)	0.01	43.3 (2.73-684.3)	0.007
2–5 years	12	0.97 (0.23-4.16)	0.97	2.2 (0.09-54.1)	0.63
>5 years	36	7.6 (2.90-19.9)	<0.001	13.1 (1.85-93.4)	0.010
CPR during intubation	56	0.09 (0.04-0.22)	<0.001	0.04 (0.01-0.23)	<0.001
Difficult airway history^e^	29	-	-	-	-
Airway bleeding	14	3.5 (1.0-12.0)	0.04	6.1 (0.50-75.2)	0.16
Airway swelling	18	13.7 (2.9-126.7)	<0.001	240.1 (13.6-4237.2)	<0.001
Trauma^e^	16	-	-	-	-
Genetic syndromes	13	0.49 (0.14-1.71)	0.27	1.5 (0.15-15.9)	0.72
Craniofacial diseases^f^	15	10.2 (2.2-47.8)	0.003	51.6 (2.50-1065.1)	0.01
Musculoskeletal diseases	11	14.8 (1.8-119.8)	0.01	4.2 (0.12-145.1)	0.43
Multiple risk factors					
No risk factors	52	1 (reference)	-	NA	
One risk factor	35	8.25 (3.0-22.7)	<0.001	NA	
Two risk factors	19	46.8 (9.0-242.8)	<0.001	NA	
More than two risk factors^e^	4	-	-		

Of the 16 RRT patients who had an identifiable risk factor, 11 (68.8%) were intubated on the first attempt. However, 2 of 16 (12.5%) were intubated on the fifth attempt. One of these patients had blood in their airway and the second had a craniofacial anomaly. Prior hospitalization and intubation records showed that both of these patients had been intubated previously without difficulty.

In multivariable logistic regression (Table 3), having a craniofacial abnormality (adjusted OR [aOR], 51.6; 95% CI: 2.50-1065.1), airway swelling (aOR, 240.1; 95% CI: 13.6-4237.2), airway trauma (all trauma patients required DART intubation), and older age (1-2 years vs. <1 year: aOR, 43.3; 95% CI: 2.73-684.3 and >5 years vs. <1 year: aOR, 13.1; 95% CI: 1.85-93.4) significantly increased odds for requiring the DART. Additionally, as the number of risk factors for a difficult airway increased so did the odds of needing the DART for all risk factors (P < 0.001). However, airway bleeding (95% CI, 0.50-75.2), musculoskeletal disease (95% CI: 0.12-145.1), and genetic syndromes without craniofacial abnormalities (95% CI: 0.15-15.9) did not significantly increase the odds of having a difficult airway that required DART management. The odds for undergoing CPR during intubation were lower in patients cared for by the DART than for those cared for by the RRT (aOR, 0.04; 95% CI: 0.01-0.23). 

Known versus unknown difficult airway history

Of patients who required the DART, 21 (42%) did not have a history of difficult airway; intubation was initially attempted by the RRT in 12 of these patients. All patients with a known history of a difficult airway had initial intubation attempts by the DART. DART patients without a history of difficult airway, that is, an unanticipated difficult airway, had risk factors similar to those of patients with a difficult airway history. There were no significant differences in genetic abnormalities (P = 0.16), airway bleeding (P = 0.20), or airway trauma (P = 0.43) between patients with and without a history of difficult airway. Patients with a history of difficult airway had more craniofacial (P < 0.001) and musculoskeletal diseases (P = 0.04) and fewer airway swelling symptoms (P = 0.04) than did patients without a history of a difficult airway (Table 4). 

**Table 4:** Comparison of patients with known history of difficult airway to patients without a history of difficult airway intubated by the difficult airway response team. ^a^Difficult airway history indicates patients who had been diagnosed by an anesthesiologist in the past. Airway bleeding includes patients with post-tonsillectomy bleeding and airway trauma. Airway swelling includes patients with anaphylaxis, airway burns or inhalational injury, subglottic stenosis, and airway trauma. Trauma includes patients who had been in a motor vehicle crash, had attempted suicide, or had a gunshot wound. Genetic syndromes include Algille’s, Epidermolysis bullosa, and Moya Moya disease. Craniofacial diseases include Treacher-Collins, Pierre-Robin, Noonan’s, and Hunter’s syndrome. Musculoskeletal diseases include muscular dystrophy. ^b^Statistical differences were assessed with univariable logistic regression modeling. ^c^Craniofacial conditions include conditions that are caused by embryologic etiologies as well as those caused by genetic mutations. Patients with genetic etiologies for their craniofacial condition were included in Craniofacial analyses only. All patients with a craniofacial abnormality had a history of a difficult airway and none were newly diagnosed.

Comorbidity^a^	Total	History of difficult airway (n = 29)	No history of difficult airway (n = 21)	P-value^b^
Age, n (%)				0.06
<1 year	13	8 (28)	5 (24)	
1–2 years	8	7 (24)	1 (5)	
2–5 years	3	0	3 (14)	
>5 years	26	14 (48)	12 (57)	
Airway bleeding, n (%)	10	4 (14)	6 (29)	0.20
Airway swelling, n (%)	16	6 (21)	10 (48)	0.04
Trauma, n (%)	16	8 (28)	8 (42)	0.43
Genetic syndromes, n (%)	4	1 (4)	3 (14)	0.16
Craniofacial diseases, n (%)^c^	8	13 (45)	0	<0.001
Musculoskeletal diseases, n (%)	9	8 (28)	1 (5)	0.04
Multiple risk factors, n				
No risk factors	8	6 (21)	2 (10)	0.29
One risk factor	21	9 (31)	12 (57)	
Two risk factors	17	11 (34)	6 (29)	
More than two risk factors	4	3 (10)	1 (5)	

## Discussion

In this analysis of our institution’s RRT and DART databases, we identified several risk factors for difficult airway management that necessitated the DART for airway securement. Risk factors included airway swelling, craniofacial anomalies, trauma, and ages 1-2 years and >5 years. The odds increased for patients who had multiple risk factors. In addition, patients with and without a history of difficulty during airway management had similar risk factors. However, congenital abnormalities were more common in those with difficult airway history. Our findings support the hypothesis that difficult airway management can be predicted in children outside the perioperative setting and led to the creation and deployment of a pediatric-specific DART and consultation service. We believe that this is the first comparison of emergency response teams to evaluate patient comorbidities and scenarios that could predict the need for a hospital-based DART with specialized personnel, equipment, and techniques.

Interestingly, genetic syndromes without craniofacial involvement did not increase the risk for DART intervention, nor did airway bleeding or musculoskeletal diseases. Similar to results published by Sterrett et al. [9], we found that intubation by both the DART and RRT occurred throughout all hours of the day and night. Not only must hospitals have RRTs staffed with expert personnel, these teams must also be readily available 24 hours a day. These findings underline an opportunity for clinicians to consult and create airway management plans for children at risk for a difficult airway prior to respiratory distress and address gaps in staffing, as advanced-level practitioners may be needed during the night. 

Patients with craniofacial abnormalities or airway swelling, or who were 1-2 years old, were significantly more likely to have airway management by the DART in our study than were children without these risk factors. These findings are consistent with previously identified risk factors for difficult airway management in children [3,9,10]. Unexpectedly, active chest compressions were more common during intubations performed by the RRT, suggesting that glottic movement did not contribute to the ease of endotracheal tube placement. Patients with airway bleeding did not have an increased requirement for the DART; however, bleeding was not quantified and was likely minimal in these patients. In contrast to literature identifying age less than one year as a risk factor for difficult intubation by anesthesia providers in the OR [2,11,12], our study showed that this age group did not need the DART more frequently than other age groups. This finding is more consistent with results from Graciano et al. [3], who showed in univariate analysis that age < 2 years was associated with difficult intubation. Unlike previously published literature, we are evaluating difficulty in emergent intubations encountered across multiple subspecialties, including neonatology, pediatric critical care, and emergency medicine. Intensivists from both the NICU and PICU routinely intubate children in this age group, suggesting that age alone does not increase the risk for difficulty during emergent intubation. 

Limiting intubation attempts in patients with a history of a difficult airway may benefit significantly from early identification and airway management planning. In our study, patients with a history of difficult airway still required a median of 2.5 attempts by the DART to secure the airway. Performing more than three direct laryngoscopic intubation attempts significantly increases the risk of complications, including hypoxia and cardiac arrest [12]; therefore, efforts to optimize first-attempt success is paramount. 

Recognizing the need to keep laryngoscopic attempts to a minimum, in 2015, our institution transitioned to having pediatric anesthesiology attendings in-house 24 hours a day, seven days per week to respond to all pediatric DART calls. In 2017, the pediatric difficult airway consult service was also developed and implemented [13,14]. Airway management may not be a priority for non-anesthesiology-trained physicians admitting children without respiratory distress to the hospital, suggesting that automated screening for patients at risk for a difficult airway may be beneficial. Anesthesiologists, pediatric intensivists, neonatologists, and emergency physicians may appreciate early identification of these patients because it would allow time to implement airway management plans and alert the DART prior to respiratory distress. 

Certain clinical scenarios can predict difficult airway management. In our study, we found that patients with traumatic injuries who presented to the ED had an increased likelihood of needing the DART. Because pre-hospital personnel alerts the ED before arrival, early identification of airway trauma can be communicated. Thus, the DART could be activated before patient arrival. Specifically, clinicians must execute extensive preparation prior to conducting airway management for patients under investigation for SARS-CoV-2 infection, even if they do not have a difficult airway history [15]. Thus, early screening and implementation of airway management plans for patients under investigation for difficult airway could optimize first-attempt success and minimize advanced airway equipment contamination and clinician exposure during a respiratory emergency.

The most common locations for pediatric intubations by both the RRT and DART were the ICUs and ED. Advanced airway equipment was also needed more frequently for DART intubations than for RRT intubations. DART carts stocked with advanced airway equipment [5] may be strategically placed throughout the hospital in higher-volume locations. The database records showed that physicians commonly used operative laryngoscopy with rigid bronchoscopes, flexible bronchoscopes, and videolaryngoscopes for emergency airway management. Therefore, personnel on the DART should be regularly trained in the use of this equipment [16]. Creating, maintaining, and processing the equipment on each cart is time-consuming and costly, and having carts at each high-volume location may not be financially feasible for all institutions. Development of one mobile cart may be more realistic for lower-volume facilities.

Limitations

There are several limitations to this study. First, because this was a retrospective analysis of a prospective database, some real-time data associated with each intubation were missing. Specifically, the rank of the physician who successfully placed the endotracheal tube was present only half of the time. Second, in some situations, the DART should have been activated but was not. One patient underwent six intubation attempts by the RRT, a number significantly greater than the maximum of three attempts that policy recommends before initiating the DART. Third, many critical care physicians who respond during RRT calls at our institution have dual training in both anesthesiology and pediatric critical care. These physicians have advanced training in airway management and may not require backup assistance from the DART; however, if many anesthesia-trained intensivists were intubating as members of the RRT, our results would have shown fewer differences between the RRT and DART groups. This added level of expertise may reduce the generalizability of our single-center study. Fourth, active CPR was performed more commonly during RRT intubation; however, whether compressions were immediately halted during endotracheal tube placement is unknown. Lastly, patients with a difficult airway history all received a DART page at the onset of respiratory distress. This practice precluded the RRT from making any initial attempts at intubation and potentially biases the results for predicting the need for the DART over the RRT in this patient group. To address this bias, we performed a comparison between patients with and without a history of difficult airway.

## Conclusions

Respiratory compromise in children can occur day or night, but patients with a difficult airway can often be predicted. Having a designated difficult airway management team in the hospital that is always available and knowledgeable in advanced airway techniques is a central component of optimizing safety for pediatric inpatients. Identifying at-risk patients and implementing airway management plans prior to respiratory distress may improve first-attempt intubation success.
